# Determination of motor activity and anxiety-related behaviour in rodents: methodological aspects and role of nitric oxide

**DOI:** 10.2478/intox-2013-0020

**Published:** 2013-09

**Authors:** Natalia Sestakova, Angelika Puzserova, Michal Kluknavsky, Iveta Bernatova

**Affiliations:** Institute of Normal and Pathological Physiology, Slovak Academy of Sciences, Centre of Excellence for Examination of Regulatory Role of Nitric Oxide in Civilization Diseases, Bratislava, Slovak Republic

**Keywords:** open field, elevated plus maze, zero maze, black and white box, anxiety, nitric oxide

## Abstract

In various areas of the bio-medical, pharmacological and psychological research a multitude of behavioural tests have been used to investigate the effects of environmental, genetic and epi-genetic factors as well as pharmacological substances or diseased states on behaviour and thus on the physiological and psycho-social status of experimental subjects. This article is reviewing the most frequently used behavioural tests in animal research (open field, elevated plus maze, zero maze, and black and white box). It provides a summary of common characteristics as well as differences in the methods used in various studies to determine motor activity, anxiety and emotionality. Additionally to methodological aspects, strain, sex and stress-related differences as well as the involvement of nitric oxide in modulation of motor activity and anxiety of rodents were briefly reviewed.

## Introduction

**Figure 1:**
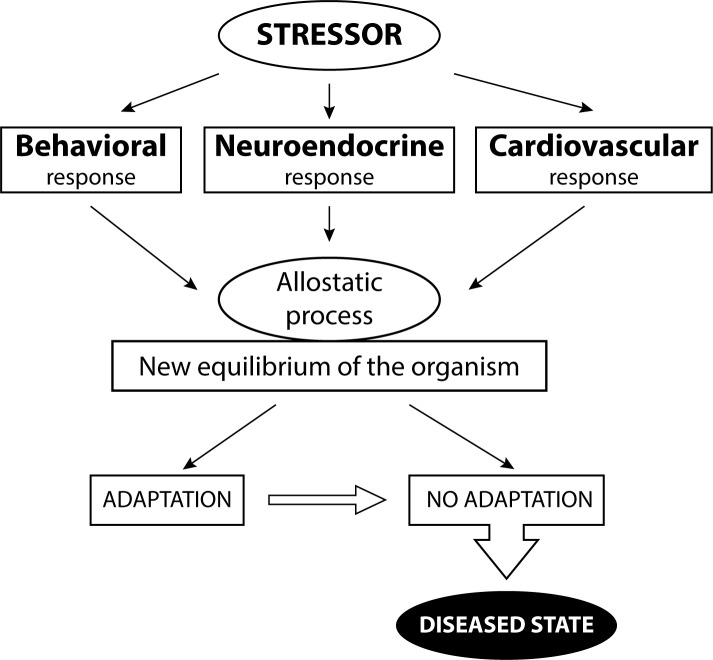
Schematic representation of a multidimensional concept of response to aversive factor (i.e. stressor). Activation of various biological systems, including neuroendocrine activation, behavioural responses and cardiovascular response, leads the organism to set up a new homeostatic state via allostatic processes. If aversive stimuli are numerous, major and/or long-lasting feedback mechanisms are incapable of restoring the new equilibrium (homeostasis) and the response of the organism (i.e. stress) becomes inadequate, which may result in various diseased states. (Modified according to McEwen, 2000; Van Reeth *et al*., 2000; Darnaudéry & Maccari, 2008).

In the twentieth century an extensive range of behavioural tests was developed in animal research. Nowadays, behavioural tests are used in many areas of bio-medical, pharmacological, toxicological and psychological/ethological research. The aim is to evaluate the effects of various factors, such as environmental challenges, genetic and epi-genetic factors, diseased states or chemical and pharmacological substances, on the physiological and psycho-sociological status of experimental subjects. As any aversive factor that disrupts homeostasis of the organism can be considered a stressor, behavioural testing is a useful non-invasive tool to determine detrimental effects of stress on the whole animal level (Figure 1).

Alterations in unconditioned spontaneous behaviour in response to an adversive stimulus may suggest functional and/or structural alterations in the central nervous system, autonomic nervous system, hypothalamic-pituitary-adrenal axis, and/or changes in the effector organs as the cardiovascular system, digestive system or skeletal muscles.

In each behavioural test, the ability of the animals to cope with the new situation is determined by analysis of alterations in their behavioural activities, such as locomotion, immobility, defecation, urination, etc. The aversive incentives used during testing may vary. The most frequently used stimuli are new environment, illumination or water environment. However, there are several sources of inter-laboratory variations in behavioural testing that may affect the results of the experiment (Table 1).

**Table 1:** Sources of inter-laboratory variation in behavioural tests.

animal species, strain, age and sex
housing conditions, light cycle, prior handling
prior test experience, number of test repetitions
adaptation to test laboratory, time of testing, illumination level
presence/absence of experimenter in test room
construction of test apparatus
definition and validation of measures

Modified according to Rodgers *et al*. (1997).

In this article we give a brief overview of the most frequently used behavioural tests, aimed primarily on investigation of motor activity, anxiety-related and emotional characteristics of rodents and methodological variability used in various laboratories. In addition to methodological aspects, strain- and sex-related differences in rodent behaviour and the involvement of nitric oxide were reviewed.

## Open field test: Equipment and procedure of testing

The most frequently used method in behavioural research is the open field test. This method was published for the first time by Hall and Ballachey in 1932 in the article “A study of the rat‘s behavior in a field: a contribution to methods in comparative psychology” as the first test to monitor anxiety-related behaviours, exploratory behaviour and emotionality in rats.

Broadhurst (1969) described the open field as a relatively standardised and reliable test. Today there is a great variability of the testing conditions available in the literature. Differences can be observed in the form of the open field arena (square, rectangular or circular), its colour, illumination and recording methods (Berton *et al*., 1997; Pardon *et al*., 2002; Chakraborti *et al*., 2008; Alstott & Timberlake, 2009; Fan *et al*., 2011).

The apparatus itself can consist either of the animal's own cage (home cage test) or of a new arena, which is much larger than the animal's home cage (novelty open field test). The usual size of the novelty open field for rats is 100×100 cm. However, some authors used smaller arenas, for example 90×90 cm (Fan *et al*. 2011), 60×60 cm (Pardon *et al*., 2002), 40×40 cm (Dubovicky *et al*., 1999; Verma *et al*., 2009) or even 25×25 cm for mice (Krishna *et al*., 2013). Similarly, the size of the circular open field varies. Francis *et al*. (1999) used circular open field sized 1.6 m in diameter, Alstott and Timberlake (2009) used an arena with 1.67 m in diameter and Bond and Di Giusto (1977) with 0.92 m in diameter. Regarding colour, usually all walls and floor of the open field are black but some authors used floor and walls painted white (Berton *et al*., 1997). Interestingly, Chakraborti *et al*. (2008) used white walls and a green floor.

Illumination is another factor that can affect the results achieved. The intensity of illumination is different according to different authors but its reduction to 7–8 lux was shown to reduce the luminosity-related component of aversion in the open field. Conversely, higher intensity (250–360 lux) is widely used to increase the animal‘s aversion to the environment (Berton *et al*., 1997; Pardon *et al*., 2002; Ramos *et al*., 2003). Unfortunately, in many studies the information on light intensity is missing, which reduces the possibility to compare individual studies.

The testing procedure itself usually starts by placement of the animal tested in the centre of the open field. However, some authors prefer to start the testing by placement of the rat in the corner of the field (Chakraborti *et al*., 2008). The activity of the animal is usually recorded for 5 or 10 min, but in some studies longer duration of testing (15–60 min) was used, depending on the experiment (Mach *et al*., 2008; Krishna *et al*., 2013; Talarovicova *et al*., 2009; Weiss *et al*., 2004). During the given time, the exploratory behaviour of rodents is determined either manually by an experienced observer or electronically. To quantify the locomotion of the rats manually, the floor of the arena can be divided into squares of 20×20 cm or 10×10 cm (Berton *et al*., 1997; Pardon *et al*., 2002; Ramos *et al*., 2003). The activity is recorded when the rat crosses the line. According to some authors, a line-crossing is counted only when the animal crosses the line with all four paws (Swiergiel & Dunn, 2007), while other authors count the activity if the animal moves both forepaws across the line (Schiller *et al*., 1991). Currently there are two electronic methods available. The activity can be determined by using photo-beams (Dubovicky *et al*., 2007; Mach *et al*., 2008) or it can be recorded by a video camera and then evaluated and analysed by videotracking software. Videotracking systems allow a continual recording of behaviour, which is more precise than manual counting. Additionally, videotracking systems allow to divide the arena virtually into central and peripheral zones as well as to determine the time spent in the corners within a peripheral zone. Then the indicators, as e.g. the total distance travelled, time spent in the central and peripheral zone, distance travelled in the central and peripheral zones, time of immobility, mean speed, maximal speed, freezing bouts, can be evaluated in all zones separately (Ramos & Mormede, 1998; Dubovicky *et al*., 2007). Additional activities which can be assessed in open field include: rearing, defecation, urination, freezing, grooming, jumping, escape attempts and vocalization (Archer, 1973; Ramos & Mormede, 1998). However, the most established indicators of emotional behaviour in the open field test are ambulation and defecation (Lister, 1990). It has been proposed that fear response (or anxiety) of the animal exposed to a new and thus potentially dangerous environment is accompanied by high defecation as well as by low ambulation, especially in the central zone (Hall, 1934; Gentsch *et al*., 1987; Bernatova *et al*., 2011). Other behavioural elements and their interpretation are described in Table 2.

**Table 2:** Variables recorded in the open field test and their interpretations.

Interpretation	Behavioural elements
Locomotion	total distance travelled, total zone entries
Vertical activity	rear frequency, rear duration, grooming
Exploration	total distance travelled, total zone entries, total entries to the central zone, total entries to the periphery zone, total entries to the corner zone
Risk assessment	total stretch attend posture, total sniffing
Decision-making	periphery zone returns, corner zone returns, grooming
Anxiety	decreased total locomotor activity, lower distance travelled in central zone, lower% of time spent in central zone, higher% of time spent in the periphery zone, in the corners

Modified according to Liebsch *et al*. (1998).

A further important issue, which has to be taken into account in behavioural research, is the number of test repetitions performed in one animal that results in reduced activity in the open field due to habituation. As described by Thompson and Spencer (1966), habituation is a form of simple nonassociative learning, in which the volume of the response to a specific stimulus decreases with repeated exposure to that stimulus. To distinguish habituation from the other nonspecific declines in behaviour, nine criteria common to various habituating responses were identified (for details see Thompson & Spencer, 1966). Habituation can be determined either in the frame of one testing session (so called within-session habituation) or in a sequence of several sessions (between-session habituation). In both cases habituation is a useful parameter in animal testing, which depends on the design of the experiment.

Another general factor that may affect the results of each study is animal handling before and during the study. Significant interactions were observed also between animal handling and housing conditions (Rasmussen *et al*., 2011; Pritchard *et al*., 2013).

### Effect of strain, sex and stress in modulation of open field behaviour

As mentioned above, the open field test has been used in many studies to investigate behaviour of rodents under the influence of various factors.

One of the obvious findings in the open field is that ambulation of female rats of different strains is usually higher than that of males (Ramos *et al*., 1997; McCormick *et al*., 2005; Bernatova *et al*., 2010). In addition to sex differences, there are studies showing significant strain-related (i.e. genetic) differences in the behaviour of rats. For example, investigation of open field activities of spontaneously hypertensive rats (SHR), Wistar-Kyoto rats (WKY), Brown Norway, Wistar Furth, Fisher 344 and Lewis rats showed that SHR and Brown Norway rats were more active, while WKY rats showed relatively low activity (Ramos *et al*., 1997). It has been suggested that higher intensity of exploration in females could be important from the evolutionary point of view. As potential mothers, females should get acquainted more profoundly with the unknown environment than males, so as to secure a quiet course of pregnancy, delivery and care of offspring (Dubovicky *et al*., 1999).

Additionally, the existence of quantitative trait loci (QTL) for emotionality-related behaviour was observed in rats (Ramos *et al*., 1999). Studies showed that the region near the locus Ofil 1 on chromosome 4 increased significantly locomotion in the central zone in female F3 rats, which originate from the mating of Lewis and SHR rats (Vendruscolo *et al*., 2006).

Besides behavioural disorders per se, altered exploration, anxiety and emotionality can be found also in many other disorders. In our previous research on behavioural aspects of hypertension we observed a positive correlation between horizontal motor activity and blood pressure in rats (Bernatova *et al*., 2011). This correlation suggests a crosstalk in the modulation of behaviour and blood pressure, supposedly via the involvement of the sympathetic nervous system. A similar hyperactivity of SHR in the open field as compared to normotensive rats was previously observed by Knardahl and Sagvolden (1979) and by Gentsch *et al*. (1987). Additionally, hyperactivity of female SHR was observed compared to Lewis females (Vendruscolo *et al*., 2006) and in borderline hypertensive females vs. Wistar females (Bernatova *et al*., 2010).

Stress, which is another important factor in the aetiology of both behavioural disorders and hypertension, also affects open field behaviour. Chronic unpredictable mild stress reduced the number of grid crossings as well as rearing and grooming behaviour in rats and prolonged the time spent in the central zone (Fan *et al*., 2011). The authors assume that the changes observed were associated with microstructural alterations due to elevation of inducible nitric oxide (NO) synthase expression in the brain, which, as has been reported, play a role in the process of stress-induced neurodegeneration. Masood *et al*. (2003) showed that restraint stress reduced ambulation and rearing of rats in the open field test. In our studies we observed delayed between-session habituation in male borderline hypertensive rats (BHR) exposed to chronic crowding. This was associated with an increase of blood pressure and of the relative adrenal gland weight (Bernatova *et al*., 2010). Dubovicky *et al*. (1999) observed that repeated stress during the neonatal period led to reduced habituation in the open field test in adult Sprague-Dawley (SD) male rats but not in females. On the other hand, chronic emotional stress in adult SD rats did not alter habituation processes (Dubovicky & Jezova, 2004). Social isolation stress was shown to affect rodent behaviour in a strain-dependent manner. Following social isolation, one of the most widely reported findings is increased locomotor activity in response to novel situations in Wistar rats (Domeney & Feldon, 1998; Weiss *et al*., 2000). On the other hand, this spontaneous behaviour was not observed in SD rats, which were shown to be more vulnerable to isolation-induced anxiety and depressive-like behaviours (Weiss *et al*., 2000). Additionally, anxiety- and depressive-like behaviour were significantly increased in social instability stressed SD females compared to non-stressed ovariectomised rats yet not in sham-operated controls, suggesting a protective role of sex hormones in the development of stress-induced behavioural disorders in females (Al-Rahbi *et al*., 2013). Thus the effect of stress on open field behaviour depends on many factors and variable results can be observed depending on protocol, strain, sex and age of the subjects tested.

Although the open field test was originally developed for comparative psychology in rodents, nowadays it can be used to determine welfare in farm animals, such as pigs (Mormede at al., 1994), chickens (Webster & Hurnik, 1989), quails (Jones at al., 1991), sheep (Moberg *et al*., 1980) and cattle (Mullens *et al*., 2006).

## Elevated plus maze: Equipment and procedure of testing

Another method often used in behavioural research is the elevated plus maze test (EPM). As EPM is suitable for investigation of anxiety, it is frequently used together with the open field test. The EPM test allows to determine anxiety-related processes which may stay undetected by other tests, since EPM includes conflict of approach and avoidance and elements of both passive and active avoidance at the same time (Montgomery, 1955; Handley & McBlane, 1993). The EPM test was introduced by Montgomery (1955) and validated later by Pellow *et al*. (1985). In his original study, Montgomery (1955) reported that rats displayed different signs of fear during exploration in enclosed or elevated alleyways.

The EPM apparatus consists of four arms in the shape of a plus sign risen above the ground from 50 cm to 100 cm (Pellow *et al*., 1985; Berton *et al*., 1997; van Gaalen & Steckler, 2000; Pardon *et al*., 2002; Verma *et al*., 2009). Two opposite arms are open while further two opposite arms are closed by usually 15–50 cm high walls (Berton *et al*., 1997; van Gaalen & Steckler, 2000; Pardon *et al*., 2002; Shum *et al*., 2005; Mällo *et al*., 2006; Verma *et al*., 2009). In the study of Pellow *et al*. (1985), no curbs of the open arms were used. Nowadays in some studies the open arms have curbs along the edges (usually 0.5–1 cm) to prevent the animal from falling (van Gaalen & Steckler, 2000; Pardon *et al*., 2002; Shum *et al*., 2005; Braun *et al*., 2011). There is a central square platform in the centre of the cross, usually of the size of 10×10 cm for rats (Berton *et al*., 1997; Pardon *et al*., 2002; Mällo *et al*., 2006) and 5×5 cm (van Gaalen & Steckler, 2000) or 6×6 cm (Shum *et al*., 2005) for mice that gives access to all four EPM arms.

The testing procedure starts with placement of the animal in the central platform of EPM facing an open arm (Berton *et al*., 1997; Pardon *et al*., 2002) or closed arm (van Gaalen & Steckler, 2000) and this lasts usually 4 or 5 min (Berton *et al*., 1997; van Gaalen & Steckler, 2000; Pardon *et al*., 2002; Shum *et al*., 2005; Mällo *et al*., 2006; Verma *et al*., 2009). Similarly to the open field test, behaviour in EPM can be determined manually or electronically. The most important variables determined in EPM are the number of entries into each arm, time spent in each of the arms and time spent in the central square. The observer can also record the number of head-dipping over the sides of the open arms as well as end-arm explorations, i.e. how many times the animal reached the distal end of the open arm (Pardon *et al*., 2002). Additional variables that can be determined include the number of line crossings, time spent in the open part, the number of approaches towards the central area, the number of open arm entries and the total number of arm entries (Mällo *et al*., 2006). Closed arm returns (it means exiting the closed arm with forepaws and immediately return into the closed arm), head dipping over the sides of the maze and stretch attend posture (SAP, stretching the head and shoulders forward and subsequently retraction to the original position) are considered to be risk assessment behaviours (Rodgers & Cole, 1993). Other behavioural elements and their interpretation are described in Table 3.

**Table 3:** Variables recorded in the elevated plus-maze and their interpretations.

Interpretation	Behavioural elements
Locomotion	total arm entries, closed arm entries, total flatback approach
Vertical activity	rear frequency, rear duration, grooming
Exploration	total head dips, total stretch attend posture
Risk assessment	total stretch attend posture, total sniffing, closed arms returns, head dippings
Decision-making	closed arm returns, grooming,% centre time,% closed time
Anxiety	total arm entries, open arm entries, % open entries,% open time,% closed time,% centre time, closed arm returns,% protected head-dipping,% protected stretch attend posture,% protected sniffing,% protected flatback approach

Modified according to Rodgers *et al*. (1997).

Similarly to the open field, EPM is based on innate aversion of rodents to open space. Treit *et al*. (1993) showed that exposure of rats to EPM for 18 consecutive days did not change their avoidance to open arms. These results suggest that the aversion to open space on the elevated plus-maze is not related to the new environment. Moreover, other experiments indicated that it is rather fear of open space than fear of heights which leads to avoidance of the open arms, because reduction of maze height (50, 25, 6 cm) did not increase open-arm activity of the rats (Treit *et al*., 1993). The study on the validity of the elevated plus-maze test carried out by Pellow *et al*. (1985) showed that rats avoided the open arms also after changing the illumination in both arms. It was also found that animals confined to the open arms for 20 min displayed more behavioural and physiological signs of fear (e.g. higher immobility, freezing, higher defecation) and higher concentrations of plasma corticosterone than animals confined to the closed arms. Nevertheless, rats confined to the closed arms also showed elevated corticosterone levels compared to the home-cage control group (Pellow *et al*., 1985). Thus open arms are more aversive for rodents than closed arms, while a certain degree of aversion is present also in the closed arms, which is associated with exposure to new environment (Ramos & Mormede, 1998). An important factor is again the number of test trials performed by one animal. File (1993) showed that in naive rats benzodiazepines manifested anxiolytic effects in the EPM test, but in rats with previous experience in EPM benzodiazepines were inefficient.

### Effect of strain, sex and stress in modulation of EPM behaviour

Similarly to open field, EPM can be used to determine the effect of various factors on fear-related behaviour of rodents. Sex- and strain-related differences were observed in various studies. In the study of Ramos *et al*. (1997) females of SHR and Lewis rats spent a longer time in the open arms than respective males. Additionally, SHR and Lewis male rats showed respectively the highest and the lowest levels of entries in the open arms, without differences either in total or in closed arm entries. No significant strain-related differences in females were observed in these parameters (Ramos *et al*., 1997). Similarly, less aversion to open arms was found in female hooded Lister rats (Johnston & File, 1991) and young SD rats (Leussis & Andersen, 2008) than in the males of the same strain. Gentsch *et al*. (1987) observed interstrain differences between SHR and WKY in EPM. According to their findings, WKY showed reduced locomotion and higher reactivity to aversive stimuli (fewer entries into open arms) as compared to SHR in EPM. Strain-related differences in EPM behaviour were observed also among 8 various mouse strains (Ducottet & Belzung, 2005). Various quantitative trait loci for anxiety-related behaviour located at various chromosomes were observed in different mouse strains (Clément *et al*., 2002). Nevertheless, strain-related differences should be presented with caution as different results can be achieved depending on conditions of testing (Clément *et al*., 2002).

In prenatally stressed rats anxious behaviour was observed in both males and females (Salomon *et al*., 2011). Zuena *et al*. (2008) found anxiogenic effect of prenatal stress in male rats yet anxiolytic effect in females. According to Wigger and Neumann (1999) neonatal stress aggravated anxious behaviour in EPM in both adult males and females, compared to the respective control group. Additionally, adolescent separation of SD rats produced both behavioural and neural changes associated with stress-related depression and anxiety, however decreased open arm time was observed only in females (Leussis & Andersen, 2008). Similarly, social isolation of SD at weaning produced an anxiogenic profile in the EPM test (reduced open arm entries) in males but not in females (Weiss *et al*., 2004). Increased anxiety in EPM was also observed in other stress models (Carnevali *et al*., 2012; Pechlivanova *et al*., 2012). On the other hand, predictable chronic mild stress in adolescence reduced depressive- and anxiety-like behaviour caused by chronic unpredictable stress in adult rats (Suo *et al*., 2013). Different authors observed that stressed animals displayed more anxious behaviour but handling decreased their fearfulness in EPM (Vallée *et al*., 1997; Schmitt & Hiemke, 1998; Gouveia *et al*., 2013).

## Zero maze test: Equipment and procedure of testing

The elevated zero maze is a variation of EPM which includes both classical and new ethological measures in the analysis of anxiety-related behaviour. The advantage of zero maze compared to EPM is that the former removes any discrepancies in evaluation of time spent in the central square of EPM (Shepherd *et al*., 1994). The design incorporates an elevated circular platform which is divided into four sections of equal length (Braun *et al*., 2011). The diameter of the maze depends on the animal strain; smaller mazes are used for mice (Heisler *et al*., 1998; Cook *et al*., 2002), while bigger mazes are used for rats (Braun *et al*., 2011). Two opposite arcs of the zero maze are enclosed by a wall (approximately 11–30.5 cm high) and other two opposite arcs are open, usually with curbs of various heights (approximately 0.25–1.3 cm) (Heisler *et al*., 1998; Cook *et al*., 2002; Parfitt *et al*., 2007; Cleck *et al*., 2008). Zero maze thus allows rodents uninterrupted exploration of the maze without turning around and thereby reducing the variability of the results (Kulkarni *et al*., 2007; Schulz *et al*., 2011). Similarly to other tests, illumination is a significant factor affecting the results achieved. Parfitt *et al*. (2007) observed that locomotion of mice in the zero maze was very low when illumination higher than 20 lux was used. The testing procedure itself starts with placement of the rat or mouse in the centre of the closed section of the maze and the animal is allowed to investigate the circular arena usually for 5 min (Parfitt *et al*., 2007; Cleck *et al*., 2008; Braun *et al*., 2011). To determine the activity of rodents both video-tracking systems and counting by trained observer can be used (Heisler *et al*., 1998; Cook *et al*., 2002; Cleck *et al*., 2008).

Similarly to EPM, the principle variable measured as an anxiety marker is the percentage of time spent in the open area. Shorter time intervals spent in the open areas are interpreted as increased anxiety (Pellow *et al*., 1985). The number of entries into closed compartments is considered an index of general activity (Rodgers & Dalvi, 1997). According to Schulz *et al*. (2011), the risk assessment behaviour (stretch-attend posture) can be calculated by analysing the frequency and duration of sniffing the open area from inside the closed area (hind paws are inside the closed track and front paws are inside the open track). The number of head dips over the edge of the open area is another marker of anxiety, with increased head dips signalising reduced level of anxiety (Rodgers & Dalvi, 1997).

Comparison of EPM and zero maze behaviour in untreated male rats showed that in the zero maze the animals spent significantly more time in the open areas, showed more head dips, had less entries into the closed area and shorter start latency. In the same study no sex-related differences in the time spent in the open area, head dips, start latency and number of entries into the closed area were observed regardless of maze (Braun *et al*., 2011). This study showed that if time spent in the central region in the EPM test was eliminated and time in the open part calculated as percentages, the results from both EPM and zero maze were essentially equal for the independent variables (anxiety indices) evaluated.

Literature reviews showed that in mice zero maze was used more frequently to determine the role of various gene modifications and to analyse the effects of pharmacological substances than to study the effect of strain or stress (Heredia *et al*., 2012; Wilking *et al*., 2012; Davis *et al*., 2013).

## Black and white box: Equipment and procedure of testing

The black and white box test (alternatively light-dark box) is an experimental procedure which was developed for testing anxiety in laboratory rodents, described originally by Crawley (1980). The apparatus consists of two chambers, one of them (approximately 2/3 of the total area of the apparatus) is made of clear or white plastic walls and is highly illuminated. The other chamber (approximately 1/3 of the total area) is painted black and either non-illuminated (Ramos & Mormede, 1998) or illuminated by a red bulb with low light intensity, e.g. 60W (Costall *et al*., 1989) or 40W (Ramos *et al*., 2003). The light and dark chamber are connected by a small passage through which the animals can move freely (Ramos & Mormede, 1998). There is a variability in the experimental conditions in this test, mainly in the size of the compartments, transition passage and illumination (Isogawa *et al*., 2003; Ramos *et al*., 2003; Sanchez *et al*., 2003). A testing session starts by placing the animal in the centre of the illuminated compartment, facing the opening to the dark compartment (Ramos *et al*., 2003; Sanchez *et al*., 2003) for a period lasting usually 5 min (Costall *et al*., 1989; Isogawa *et al*., 2003; Ramos *et al*., 2003). The floor of both compartments is divided into squares in order to determine locomotion (Ramos *et al*., 2003). Horizontal and vertical activity can be recorded also by a photocell inside the test box (Sanchez *et al*., 2003). The parameters measured include total horizontal and vertical activity separately in each compartment, time spent in the white compartment and number of transitions between the black and white compartments (Crawley, 1981; van Gaalen & Steckler, 2000; Ramos *et al*., 2003; Shum *et al*., 2005; Salim *et al*., 2010). The major indices of anxiety vary among the studies.

### Effect of strain, sex and stress in modulation of zero maze and black and white box behaviour

As written above, zero maze and black and white box are used less frequently than open field and EPM to determine the effects of stress or sex- and strain-related differences in mice and rats. In a study investigating the effect of prenatal stress on offspring of stressed dams in SD rats using zero maze revealed increased anxiety behaviour only in female offspring (Schulz *et al*., 2011). Similarly did restraint stress decrease significantly the time spent in the open part of the zero maze, head dips and closed area entries and increase significantly start latency, with no differences observed in EPM and zero maze behaviour of male and female SD rats (Braun *et al*., 2011).

Significant strain-related differences were observed in black and white box measures (Ramos *et al*., 1997; Rex *et al*., 1999; Ramos *et al*., 2003). Ramos *et al*. (1997) have suggested that SHR and Lewis rats are a powerful tool for studying anxiety-related behaviour, with significantly lower anxiety observed in SHR vs. Lewis strain. Another study showed that male Fischer and Lewis rats displayed similar anxiety-related behaviours in the black and white box, however higher locomotor activity was seen in Fisher rats in the open field test (Chaouloff *et al*., 1995). Additionally, Fischer rats displayed a more pronounced fearful behaviour in the black and white box compared to Wistar-Harlan rats (Rex *et al*., 1999).

In the study of Ramos *et al*. (2003), significant sex-related differences were observed only in locomotion in the black compartment with higher activity in females but not in other measures determined. Henniger *et al*. (2000) showed similar behavioural differences between high anxiety-related behaviour and low anxiety-related behaviour of Wistar rat lines regardless of sex.

## Involvement of nitric oxide in animal behaviour

Nitric oxide (NO) is a well-known neurotransmitter and neuromodulator. Significant NO production was determined in various parts of the CNS (Steinert *et al*., 2010). NO is implicated in the regulation of excitability and firing, in long-term potentiation and long-term depression as well as in memory processes (Prast & Philippu, 2001). Additionally, NO was shown to be involved in modulation of motor activity and anxiety-related behaviour, yet considerable variability of its role can be found in the literature. The common way of investigation of the role of NO in modulation of animal behaviour is the use of NOS inhibitors.

Regarding motor activity, reduction of spontaneous locomotor activity by NOS inhibitors was observed by Del Bel *et al*. (2002). In our studies, chronic treatment with N^G^-nitro-L-arginine methylester (L-NAME, non-selective inhibitor of nitric oxide synthase) in the dose of 40 mg/kg/day for 4 weeks reduced locomotor activity and exploration as well as sniffing, cleaning and defecation in male Wistar rats, simultaneously with reduced NO synthase (NOS) activity in the cerebellum, cerebral cortex and thoracic spinal cord (Halcak *et al*., 2000). Moore *et al*. (1991) showed that a high dose of L-NAME (600 mg/kg) had a nearly sedative effect in rats. Moreover, NOS inhibitors N^G^-nitro-L-arginine (L-NOARG), L-NAME and 7-nitroindazole (7-NI, a relatively selective inhibitor of neuronal NOS) induced catalepsy in mice and rats (Echeverry *et al*., 2007; Lazzarini *et al*., 2005; Del Bel *et al*., 2004). In studies of Volke *et al*. (1997), 7-NI (10 mg/kg) produced a clear sedative effect in the open field test in rats while higher doses of 7-NI (80–120 mg/kg) were required to produce a similar effect in mice. On the other hand, 7-NI in the dose of 30 mg/kg failed to affect open field behaviour in rats (Hölscher *et al*., 1996). In contrast to pharmacological inhibition of neuronal NOS (nNOS), male mice lacking the neuronal NOS gene, nNOS knock-outs, showed higher frequency of entries into the centre, longer time spent in the central zone and reduced immobilisation (Kirchner *et al*., 2004). In addition to these studies dealing with reduction of NO production, Da Silva *et al*. (2000) found that L-arginine (L-Arg), a substrate of NO synthesis, did not change locomotor activity in the open field. In our studies in which WKY and BHR rats were used, differences in their open field behaviour did not correlate with NOS activity in the hypothalamus and cerebellum (Sestakova *et al*., 2011).

The involvement of NO in the anxiety-related behaviour determined in EPM was also investigated in both rats and mice. However, there are contradictions regarding the anxiolytic or anxiogenic effect of NO.

The NO synthase inhibitor L-NOARG (30–120 mg/kg) reduced the number of entries into open arms and the time spent on them in rats. These doses however decreased also the number of entries into closed arms (except 30 mg/kg). Interestingly, when the animals were tested after chronic L-NOARG administration, these effects disappeared (De Oliveira *et al*., 1997). Similarly, the number of entries into open arms, time spent on open arms, and percentage of open arm entries were reduced after acute L-NOARG administration (2 µg) into the brain of rats (Monzón *et al*., 2001). Furthermore, acute L-NAME (12.5–50 mg/kg) had an anxiogenic-like profile, as indicated by dose-dependent reductions in the time spent on the open arms, open arm entries, and the percentage of open arm entries (Vale *et al*., 1998). In addition, Kirchner *et al*. (2004) found that the number of entries into closed arms was significantly higher in nNOS knock-out male mice as compared to the control group, which was consistent with their overall higher activity. In contrast to these studies, there is a number of studies suggesting opposite effects of NOS inhibitors.

For example 7-NI significantly increased the time spent on the open arms and the percentage of entries into them in Wistar male rats in a dose-dependent manner, with a minimal dose of 40 mg/kg (Volke *et al*., 1997). In mice, 7-NI had also an anxiolytic effect in EPM, yet higher doses (80–120 mg/kg) were required to reach the same effect (Volke *et al*., 1997). Acute administration of L-NAME (10 and 60 mg/kg) prolonged the time spent on the open arms in rats. The same study showed that both short- and long-term administration of L-NAME inhibited NO production in endothelial cells and in the central nervous system and led to an increase of mean arterial pressure and decrease of NO synthase activity in brain tissue (Faria *et al*., 1997). It is however unlikely that the anxiolytic effect of L-NAME in EPM was associated with accompanying hypertension because no changes in EPM behaviour were observed in non-pharmacological two-kidney one-clip model of hypertension (Faria *et al*., 1997). In the study of Spiacci *et al*. (2008), dual effects were observed with NOS inhibitors L-NAME and 7-NI in both the EPM and forced swimming tests. While low doses of L-NAME (25 nmol) or 7-NI (1 nmol), microinjected into the brain, induced a selective increase in EPM open arm exploration and decreased immobility time, high doses (L-NAME 400 nmol, 7-NI 10 nmol) decreased locomotor activity. In the same study, L-Arg (100 and 200 nmol) produced an anxiolytic-like effect in the EPM test.

Regarding the role of NO in stress-induced neurobehavioural effects, Masood *et al*. (2003) observed also a dual effect of L-NAME. The authors showed that a higher dose of L-NAME (50 mg/kg) aggravated restraint stress-induced alterations in EPM while a lower dose (10 mg/kg) attenuated them. Interestingly, 7-NI (10 and 50 mg/kg) failed to significantly affect the above mentioned stress-induced behavioural changes (Masood *et al*., 2003).

The effect of NOS inhibitors in black and white box behaviour was investigated mainly in mice. Subcutaneous L-NAME administration (25 and 50 mg/kg) reduced the time in the light box as well as the number of transitions (Czech *et al*., 2003). In mice, 7-NI (80–120 mg/kg) evoked an anxiolytic-like profile in the black and white box and the doses required to reach the same effect as in rat models were higher (Volke *et al*., 1997). Interestingly, Wultsch *et al*. (2007) observed no significant differences in nNOS knock-out mice compared to their respective wild types in light-dark box behaviour.

## Conclusion

On balance then, this literature review showed that although behavioural testing in the open field and EPM is commonly included in many bio-medical studies, many differences in methodology can significantly affect the results achieved in various laboratories. Thus detailed description of the methods used and conditions of testing is essential in behavioural research.

Regarding the involvement of NO in modulation of spontaneous motor activity and in anxiety-related behaviour of rodents, the above mentioned studies suggest a respective role of NO in the open field test and in EPM behaviour. However, the effect of NO synthase inhibitors was found to depend on the nature and dose of the inhibitor used and on the duration of the treatment. These findings call for additional research to identify if physiological levels of NO are associated with anxiogenic or anxiolytic behaviour in rodents in studies performed without NOS inhibitors.
